# Analysis of public policies to combat COVID-19 in the state of Paraná, Brazil

**DOI:** 10.3389/fpubh.2024.1384561

**Published:** 2024-07-17

**Authors:** Bruna Regina Bratti Frank Terre, Beatriz Rosana Gonçalves de Oliveira Toso, Luiz Fernando Reis, Jerry Adriani Johann

**Affiliations:** ^1^Centro de Ciências Biológicas e da Saúde, Programa de Pós-graduação Stricto Sensu em Biociências e Saúde, Universidade Estadual do Oeste do Paraná, Cascavel, Brazil; ^2^Pró-Reitoria de Pesquisa de Pós-graduação, Programa de Pós-graduação Stricto Sensu em Engenharia Agrícola, Universidade Estadual do Oeste do Paraná, Cascavel, Brazil

**Keywords:** COVID-19, public policy, health indicators, public administration, pandemic

## Abstract

**Introduction:**

The COVID-19 pandemic had a great impact on several public sectors worldwide, requiring the implementation of public policies in an organized way to contain and control the disease. Thus, this study aimed to analyze public policies to face the COVID-19 pandemic in the State of Paraná, Brazil.

**Methods:**

This was a cross-sectional, retrospective, quantitative survey carried out with data from March 2020 to March 2022 in the twenty-two municipalities that host the local health regions. Data collection was documentary, carried out from the municipal *Portal da Transparência* website, which is dedicated to making public all expenditures, and epidemiological bulletins released by the Health Department of the state of Paraná. The variables analyzed were New Cases and Deaths, Mortality and Lethality Coefficient, Incidence Rate, Vaccination Coverage, Number of Hospital and ICU beds exclusive to COVID-19, Settled Expenses destined for COVID-19 and coping measures, namely, the Declaration of Public Health Emergency, Curfew, Mandatory use of masks, Businesses closure, Teleworking of risk groups, and Suspension of activities with crowds and of classes. After collection, data underwent descriptive analysis, and subsequently, the correlation of variables was analyzed using the Spearman test. Multiple linear regression was applied using the variable selection method called *best subset selection* (BSS). The dependent variables listed were incidence rate, new cases and new deaths.

**Results:**

The results showed that coping measures, as well as the application of resources for the pandemic, were implemented heterogeneously in the municipalities studied, and the progression of the disease, the distribution of beds and vaccination occurred unevenly and may be a reflection of the limited Brazilian national governance. An important correlation was observed between the incidence rate and new deaths with vaccination coverage. In addition, the regression model showed that measures such as mandatory use of masks, closure of shops, suspension of classes, and curfew showed important correlations with the variables incidence rate, cases, and new deaths.

**Discussion:**

The study highlighted the importance of carrying out a robust analysis of public policies to face emergencies of global importance so that government entities are prepared for future crises of great repercussions, such as the COVID-19 pandemic.

## 1 Introduction

On January 30, 2020, the infectious disease COVID-19 was declared a Public Health Emergency of International Concern (PHEIC) by the World Health Organization in view of its accelerated rate of contamination, reaching several countries around the world. On March 11 of that year, the disease was classified as a pandemic, causing the WHO to alert countries to adopt coping strategies, explaining planning guidelines to support the preparation and response of countries around the world (1).

Among the initial actions recommended by the WHO, implemented by government entities through public policies, the following were listed: the mandatory use of masks, the suspension of activities with crowds and school activities, the isolation and quarantine of suspected cases, businesses closure, and social distancing, among others, and – after a year of the pandemic – vaccination against COVID-19 (1, 2) as the main policy for fighting the disease.

The study of public policies is seen as an essential tool for understanding the attribution of states and the types of interventions in society, whether in the economy or in the organization and provision of public services, as well as in the allocation of resources for this purpose (3). In the context of a global pandemic, marked by the search for actions to prevent, control and mitigate the effects generated by COVID-19, this tool has become indispensable.

At the governmental level, the integration of public policies corresponds to the most advanced stage of horizontal coordination, in which each choice and each decision must consider the effects not only in relation to the common objective that is intended to be produced but also the entire system of actions, programs, and organizations involved in achieving this objective (4). To this end, coherence in the implementation of strategies to face the pandemic among government entities has become fundamental, a fact that had not been observed by Brazil, one of the countries most affected by the disease and which presented weaknesses in national governance.

For this implementation of actions to materialize, the proper allocation of resources to various sectors, such as social assistance, economy, education and, primarily, to health – considering the organization of services with the hiring of health professionals to attend to the demand, opening of new hospital and intensive care unit (ICU) beds, among others, to avoid the collapse of health systems worldwide and, specifically in Brazil, the Brazilian Public Health System (SUS) – proved to be essential. However, the transfer of resources across the country was small compared to other countries that were successful in combating COVID-19, especially for health (5).

Both the organization in the implementation of public policies for the COVID-19 pandemic and the appropriate allocation of resources for this purpose should consider the analysis of the progression of the current epidemiological situation. In this regard, the measurement and monitoring of health indicators have become a unique tool for the policy to combat COVID-19 throughout the country, especially in the state of Paraná, as the object of the present study. To this end, this study aimed to analyze public policies to combat the COVID-19 pandemic in the state of Paraná.

## 2 Materials and methods

This was a cross-sectional, retrospective study with a quantitative approach that analyzed the implementation of public policies for COVID-19 in the state of Paraná. The study is the result of ongoing doctoral research.

Data collection was documentary and took place from secondary data released by Epidemiological Bulletins of the Secretary of Health of the State of Paraná (SESA/PR) and by the Municipal *Portal da Transparência* websites of the twenty-two (22) municipalities where the Health Regions (RS) are located over 24 months – March 2020 to March 2022. The host cities of the RSs were chosen as a study sample because they are the political-administrative health centers of the state.

After collecting the data, the variables to be analyzed were listed and divided into four categories:

- Epidemiological variables: number of New Cases (NC) and New Deaths (ND) of COVID-19; Cumulative Incidence Rate (IR), represented by the ratio between the number of confirmed cases of COVID-19 and the resident population, multiplied by 100,000; Lethality Coefficient (LC), calculated by the ratio between the number of deaths from COVID-19 and the number of registered cases multiplied by 100; Cumulative Mortality Coefficient (MC), measured by the ratio between the cumulative number of deaths from the disease and the resident population, in 100,000; Vaccination Coverage (VC), represented by the percentage of individuals vaccinated with a complete vaccination schedule – individuals with the first and second doses or a single dose – according to the resident population (6). It is important to highlight that the VC variable only presented data from January 2021, the beginning of the National Immunization Campaign against COVID-19;
- Organizational Variables: Number of hospital beds in the Brazilian Public Health System (SUS) intended exclusively for COVID-19 per 100,000 inhabitants (HOSPB) and number of beds in the SUS Intensive Care Unit exclusively for COVID-19 per 100,000 inhabitants (ICUB);
- Budgetary Variables: Total expenses incurred exclusively for dealing with the COVID-19 pandemic (EXP) and for 100,000 inhabitants (EXP1);
- Political Variables (Actions): Corresponding to the coping measures adopted by the municipalities. Among the main measures advocated by the federal and state governments, which were used for the analysis, the following were listed: (1) Declaration of Emergency in Public Health of National Importance (PHENC); (2) Businesses closing (BC); (3) Suspension of mass gathering events (SMG); (4) Suspension of classes (SLC); (5) Mandatory use of masks (MASK); (6) Telework for risk groups (TEL); and (7) Curfew (CUR). For each policy variable, a value of 0 was adopted – municipality did not adhere to the policy; and 1 – municipality adhered to the policy. From this weight, the municipalities presented a weight between 0 and 7.

After tabulation, a descriptive analysis of the data was carried out, with measurement of the mean and standard deviation of the results. In addition, the correlation between the variables was analyzed using the Spearman test, with the magnitude of the correlation effect between the variables being represented by the correlation coefficient (r_s_), which takes values between −1 and +1. Positive coefficients (_rs_ > 0) indicate a direct correlation between the variables, and negative coefficients (r_s_ < 0) indicate an inverse correlation. The correlation can also be classified as null or nonexistent (r_s_ = 0), weak (between 0.001 and 0.299), moderate (0.300 and 0.599), strong (0.600 and 0.899), very strong (0.900 and 0.999), and total (r_s_ = 1, 7).

Then, statistical analysis was performed using multiple linear regression and the variable selection method called *best subset selection* (BSS), which analyzes the regression models in all combinations of variables and chooses the one that best fits the model based on predefined parameters (7).

Linear regression is a model in which a numerical variable is estimated by the value of other variables that can be numerical or categorical. The variable to be estimated is called the dependent variable, while the others are independent. As a result of the BSS method, the variables listed as dependent were incidence rate, new cases and new deaths, which were analyzed separately. Each adjusted linear regression model has only one dependent variable, so three models were built, one for each dependent variable. Although all other variables were treated as independent, only the most relevant ones were selected using the so-called *stepwise method,* which explores a restricted set of models based on the *Akaike information criterion* (AIC). This criterion measures not only the quality of the model but also its simplicity and is used to define which variables can be excluded from the model without causing loss of information (8). This selection was made using the R MASS library.

The linear regression model, as it is a parametric method, needs to follow some validity assumptions to have statistical significance. The residuals must have an approximately normal distribution and must have a similar variance throughout the model. Furthermore, there must be a linear relationship between the dependent and independent variables, and finally, there cannot be multicollinearity, which is the presence of correlation between two or more independent variables. If independent variables are correlated, they have similar information, so only one of them must be used in the model so that information is not distorted. Therefore, the requirement for an adequate multiple linear regression is the absence of multicollinearity (9).

In this sense, regarding the adequacy of the multiple linear regression model, the absence of multicollinearity was verified with the analysis of Variance Inflation Factor (VIF), in which multicollinearity in the data is detected when a VIF value above the cutoff point is obtained, which is usually equal to 10 (10). Residual normality was analyzed using the Shapiro Wilk test, and homoscedasticity using the Goldfeld-Quandt test. To assess the absence of serial correlation, the Durbin-Watson test was used. The absence of influential points was verified using Cook’s distance, which evaluates the influence of each observation by varying the coefficients when considering regression models with and without it, adopting the cutoff point = 1, that is, the point it is considered influential if its distance from Cook is greater than 1 (11). Furthermore, the goodness of fit was validated by the model’s R^2^ as well as with the aid of the half-normal graph and simulated envelope from the hnp library of R (12).

## 3 Results

Supplementary Table S1 presents the results of the descriptive analysis of the variables analyzed in the twenty-two municipalities (22) that are home to the RSs, over a 24-month period. Regarding the distribution of SUS beds in wards, all per 100,000 inhabitants, four municipalities stood out: Ivaiporã (134.3), Guarapuava (42.0), Telêmaco Borba (38.6), and Umuarama (34.9). In addition, five other municipalities, Cornélio Procópio (26.8), Jacarezinho (24.8), Pato Branco (23.5), Maringá (22.9), and Foz do Iguaçu (22.0), presented an average number of hospital beds above the general average (21.5).

For the variable SUS beds in ICUs per 1,000,000 inhabitants, ten municipalities had values above the average (16.3): Ivaiporã (63.3), Telêmaco Borba (39.4), Francisco Beltrão (22.6), Cornélio Procópio (22.5), Cascavel (22.0), Foz do Iguaçu (21.6), Umuarama (19.6), Campo Mourão (19.4), Londrina (18.8), and Curitiba (16.4).

Maringá, Paranaguá, and Irati were the municipalities with the highest action implementation average (5.2), followed by Pato Branco, Umuarama, Jacarezinho and Cianorte (5.1), and União da Vitória, Campo Mourão and Cornélio Procópio (with average of 5.0). In other words, these municipalities had an average of 5 actions in force monthly over the 24 months evaluated. The results showed that actions such as PHENC, MASK, and TEL were adhered to by all municipalities in most of the period (average of 1). In addition, actions such as the suspension of activities with crowds and of classes were in effect for an average of 60 and 70% of the period and in the municipalities evaluated, respectively, with Jacarezinho and Guarapuava (suspension of activities with crowds of 80% of the period), and smaller municipalities such as Campo Mourão and Pato Branco (80% suspension of classes) being highlighted. In contrast, the businesses closure was implemented in an average of 20% of the evaluated period, with the municipality of Francisco Beltrão standing out with the highest average (0.4).

In the descriptive analysis of health indicators, all calculated per 1,000,000 inhabitants, the municipality of Curitiba stood out with the highest average number of new cases and deaths per month (9839.8 and 365.5, respectively). In relation to IR, municipalities such as Telêmaco Borba (12794.3), Toledo (11487.9), and Foz do Iguaçu (11345.4) stood out with the highest averages of incidence, and the general average of this indicator in the evaluated municipalities was 9125.8. Telêmaco Borba and Foz do Iguaçu were also the municipalities with the highest average of MC (246.0 and 240.1, respectively), while the general average of mortality was 172.5. For the variable LC, the municipalities of Apucarana (mean of 4.3%), Ivaiporã (4.0%), and Curitiba (3.3%) had the highest means of monthly Lethality over the 24 months of the study.

Regarding the VC variable, a monthly average of 38.5% of vaccinated individuals was observed in the 22 municipalities evaluated throughout the study periods, with emphasis on the municipalities of Maringá (46.9%), Ivaiporã (46.0%), Curitiba (43.0%), and Foz do Iguaçu (41.9%), which had the highest monthly averages. It must be considered that the VC results represent the global average over the 24 months evaluated and not the final vaccination coverage of the evaluated period, as vaccination against COVID-19 only started in January 2021.

The EXP1 variable showed that Curitiba had the highest average of settled expenses destined exclusively for COVID-19 over the period evaluated, with an amount of BRL 3,239,946.3/100,000 inhabitants, followed by municipalities such as Foz do Iguaçu (BRL 2,184.728.2/100,000), Campo Mourão (BRL 2,006,443.1/100,000), and Pato Branco (BRL 1,756,728.3/100,000/100,000).

After descriptive analysis, it was observed that the PHENC variable remained with a score = 1 in the 22 municipalities over the 24 months; that is, the PHENC declaration was carried out by all study locations and remained in force until the end of March 2022. For this reason, the variable was not considered for the other analyses.

The correlation matrix between all study variables is shown in Supplementary Table S2. It was observed that the variables NC, ND and EXP1 showed a strong/very strong positive correlation with each other (NC and ND with *r_s_* = 0.94; NC and EXP1 with *r_s_* = 0.85; EXP1 and ND with *r_s_* = 0.97); that is, municipalities presenting a high number of new cases also presented a high number of new deaths and settled expenses, demonstrating that the increase in the transfer of resources was influenced by the severity of the epidemiological situation (increase in the notification of cases and new deaths).

Furthermore, a moderate/strong positive correlation was observed between the variables ICUB and MC and ICUB and RI (*r_s_* = 0.56 and 0.64, respectively), indicating an increase in the number of beds as mortality and incidence increased, while the correlation between ICUB and CUR was negative and moderate (*r_s_* = − 0.45); that is, with the Curfew measure in effect, there was a decrease in the number of ICU beds. This fact may suggest that the curfew influenced disease control, meaning that there was not such a high demand for ICU beds.

Positive correlations were also observed between VC and EXP1+ and VC and LC (*r_s_* = 0.55 and 0.52, respectively), demonstrating an increase in VC when there was an increase in the transfer of resources to fight the disease. In addition, the increase in the lethality indicator was related to the increase in vaccination of the population. Positive correlations were also observed between VC and MASK (*r_s_* = 0.49) and between ICUB and TC (*r_s_* = 0.50), demonstrating an increase in the implementation of the businesses closure measure, while the number of ICU beds increased. In contrast, a moderate negative correlation (*r_s_* = 0.50) was observed between LC and IR, which may indicate that while the incidence increased and more people were exposed to COVID-19, the lethality of the disease decreased. This fact was also influenced by vaccination (due to the positive correlation shown above).

Tables 1–3 present the results of the multiple linear regression model of the dependent variables: Incidence Rate, New Cases and New Deaths with their respective significant factors, in sequence. The variables EXP1 and NC were not used in the model, as they presented *r _values_* above 0.9 with the ND variable. This decision was supported by the find correlation function of the caret library (13), which defines which variables should be removed, choosing those that have high correlations with the others. It is worth noting that the values of the IR variable were divided by 1,000 to adjust the values with the other variables.

**Table 1:** Significant factors in the multiple linear regression model to explain the Incidence Rate variable.

Variables	Coefficient	Standard error	Confidence interval	*p*-value
ICUB	0.138 ***	0.030	0.072–0.204	0.001
VC	0.263 ***	0.085	0.079–0.447	0.008
LC	−1,421 ***	0.292	−2.049 to −0.793	<0.001
ND	−0.006 **	0.002	−0.013 to −0.0003	0.039
BC	6,319	4.177	−2.640 to 15.279	0.153 ^ns^
SLC	4,184 *	2,258	−0.660–9.029	0.085 ^ns^
MASK	−31,276 ***	9,561	−51,784 to −10,768	0.006
Comments	22	22	22	22
R^2^/R^2^ adjusted	0.860/0.790			

Source: Prepared by the authors, 2023. *ns, not significant; ** for 5%; *** for 1%.

**Table 2 tab2:** Significant factors in the multiple linear regression model to explain the New Cases variable.

Variables	Coefficient	Standard error	Confidence interval	*p*-value
ICUB	−38,395	29,402	−101,065–24,273	0.211 ^ns^
LC	648,210 **	277,984	55,701–1240,720	0.034
IR	483,592 ***	149,889	164,109–803,074	0.006
ND	33,264 ***	2,409	28,127–38,400	<0.001
BC	−7,665,698 **	3,525,707	−15,180,566 to −150,830	0.046
SLC	−3,957,817 **	1736,248	−7,658,543 to −257,092	0.038
Comments	22	22	22	22
R^2^/R^2^ adjusted	0.940/0.916			

Source: Prepared by the authors, 2023. *ns, not significant; ** for 5%; *** for 1%.

**Table 3:** Significant factors in the multiple linear regression model to explain the New Deaths variable.

Variables	Coefficient	Standard error	Confidence interval	*p*-value
VC	−0.052 *	0.028	−0.113–0.008	0.084 ^ns^
LC	−0.305 *	0.165	−0.662–0.051	0.087 ^ns^
MC	0.020 ***	0.003	0.012–0.027	<0.001
IR	−0.402 ***	0.069	−0.553 to −0.251	<0.001
NC	0.001 ***	0.00003	0.001–0.001	<0.001
BC	5,866 ***	1.505	2.614–9.117	0.002
TEL	−1,876	1.511	−5.142–1.389	0.236 ^ns^
CUR	0.983 *	0.457	−0.004–1.972	0.051 ^ns^
Comments	22	22	22	22
R^2^/R^2^ adjusted	0.996/0.993			

Source: Prepared by the authors, 2023. * ns: not significant; ** for 5%; *** for 1%.

For the variable RI (Table 1), the model presented an adjusted R^2^ of 0.79 (*p* value <0.001), that is, the adjusted regression model explains approximately 80% of the variation in IT. With the exception of TC and SLC, all other variables were statistically significant (p value <0.05). The ICUB and VC coefficients were positive, which indicates that in the municipalities where there was a higher incidence, an increase in the number of ICU beds and higher vaccination rate was also observed, which suggests a positive response plan in relation to the worsening of the epidemiological scenario.

In contrast, the coefficients presented by the variables LC, ND, and MASK were negative. In other words, municipalities with low incidence had a higher lethality and, consequently, a greater number of new deaths; in addition, these locations had a greater effectiveness in the implementation of the mandatory use of masks.

Regarding the multiple linear regression result to explain the NC variable, the adjusted R ^2^ value was 0.91 with a *p* value <0.001. The variables associated with the increase in NC (coefficients that were positive) were LC, IR and ND, that is, the extent to which there was an increase in the number of new cases. There was also an increase in lethality (reflection of the increase in new deaths) and incidence as a consequence of the increase in cases. In contrast, it was observed that the implementation of measures such as closing shops and suspending school activities (variables TC and SLC) showed a significant reduction in the notification of new cases (Table 2).

Finally, the multiple linear regression model for the ND variable detected the presence of a possible *outlier*, as it presented a standardized residual above 3. Taking Cook’s distance test into account, it was found that this was an influential point. To adjust the analysis, the Box–Cox transformation was adopted based on the square root, which consists of extracting the square root of the values of the response variable (Box; Cox, 1964). After the transformation, the variables were selected again by the *stepwise method*, and the final model presented an adjusted R^2^ of 0.99 with a *p* value <0.001 (Table 3).

The MC and NC variables are positively associated with the increase in ND; that is, the increase in new deaths was influenced by the increase in new cases, in addition to influencing the increase in the mortality indicator. A positive coefficient was also observed for the HR variable, which may suggest greater implementation of the businesses closure measure as a consequence of the increase in the number of notified deaths. In relation to the IT variable, the coefficient was negative, and the increase in the number of deaths was related to the decrease in incidence.

The VC, LC, TEL and CUR variables were not statistically significant (*p* value >0.005). However, VC and CUR showed *p* values of 0.08 and 0.05, respectively. Although they were not shown to be significant at a 95% confidence interval, they were found to be very close to it. In the case of VC, for example, the relationship with ND showed a negative coefficient; that is, the increase in the number of deaths is related to low vaccination coverage. In addition, the increase in the number of new deaths showed an increase in the measure of the curfew in the municipalities, suggesting the implementation of containment measures as the epidemiological situation worsens.

## 4 Discussion

The COVID-19 pandemic brought numerous social, cultural, economic and, notably, political repercussions around the world, especially in Brazil. This reality also reached the Brazilian states and municipalities, and the implementation of actions and the organization of public policies occurred differently among subnational entities, as observed in national studies (14).

For example, among the implemented actions, it was observed that the closure of shops, curfew, and the suspension of mass gathering activities were the actions with the lowest average over the period, as well as the greatest variation among the evaluated municipalities. These restrictive measures were considered of paramount importance for controlling the health crisis in several territories, especially in countries that were successful in responding to COVID-19, such as China, South Korea, Canada, and Germany (15). Mass gathering activities were considered the main ways of spreading the virus (16) and, along with this, physical distancing reinforced by the closure of shops and the curfew became essential. Thus, it was expected that these measures would have better adherence among the municipalities studied.

Studies revealed that measures to restrict the movement of people were timid throughout the national territory and, generally, adopted by subnational governments. The decision to close of shops, therefore, depended on the positioning of states and municipalities, which often clashed with the recommendations of the national government at that time, which was contrary to lockdown measures (17, 18).

In an experimental study carried out by Oliveira (19) which evaluated out an evaluation of the strict restrictive measures during the pandemic in Rio Grande do Sul/Brazil, it was identified that the adoption of this type of measures essentially depends on the particularities of each location and reflects that, for these measures to be effective, they must be based on rigorous cost–benefit analyses. It was observed that strict measures adopted in advance of the outbreak or very close to its peak were unable to prevent the spread of the disease and alter its trajectory in that state.

Corroborating this aspect, another study that evaluated strategies for relaxing measures in Rio de Janeiro/Brazil, concludes that, in situations of epidemic control, relaxation measures carried out gradually are more effective in controlling the crisis than abrupt measures (20). Based on these studies, it is clear that coordination of actions must be based on systematic planning of actions for the real effectiveness of public policies.

Linear regression results showed greater adherence to businesses closure when there was an increase in the number of cases and new deaths registered. In addition to this action, the curfew and suspension of classes showed a significant correlation with the control of new cases and deaths. Furthermore, it was possible to observe that the mandatory use of masks was related to the low incidence of COVID-19. Research carried out in Brazil to describe adherence to the use of masks identified that 97.9 per cent of the local population preferred to wear a mask when leaving home (21), which corroborates the findings of the present study.

These findings confirm the importance of these actions in controlling the crisis and demonstrate that the early implementation of relaxation measures and the uncoordinated adherence to actions among government entities only aggravated the epidemiological situation, as identified in the other studies mentioned above. A study carried out by the Oswaldo Cruz Foundation (22) in an assessment of the first two years of the pandemic confirms this reality.

The present study demonstrated that the implementation of the vaccination policy against COVID-19 also occurred unevenly among the evaluated municipalities, in view of the variation in the average vaccination coverage over the study period. The results show that municipalities with higher averages of vaccination coverage demonstrated low incidence but high global averages of Lethality and Mortality.

The justifications for the disparities identified in relation to morbidity and mortality indicators may be related to different aspects that should be highlighted, including the date of onset of the disease in the municipalities and regions, demographic and population density, age distribution and characteristics of the population, health services conditions, etc. (23).

The first year of the pandemic was marked by a lack of case reporting and lack of diagnostic tests, which may have compromised the accuracy of health indicators and influenced the excess of deaths and the actual number of cases of the disease (22, 24, 25). Initial problems such as inadequate transport, difficulties in standardizing the tests performed, the type of methodology used to analyze the tests and detect the ideal time for sample collection, and the accuracy of the results made the initial diagnostic confirmation of COVID-19 a challenge worldwide (26, 27), which led to underreporting of disease data.

On the other hand, the early loosening of measures to deal with COVID-19, especially during school holidays and recess, which was identified throughout the national territory (18), culminated in a high increase in the number of deaths, compromising national health systems, with high occupancy rates of ICU beds. This period was marked by the collapse of the health system, with the occurrence of localized health crises, lack of equipment and supplies for the ICU, and depletion of the health workforce, compromising health care throughout the country (22). As a reflection of this scenario, there was a shortage of diagnostic testing, compromising the recording of the actual number of cases of the disease and, consequently, the lethality and mortality indicators.

The second year of the pandemic was marked by the start of the National Vaccination Campaign against COVID-19. The advance of vaccination in the country occurred slowly and unevenly, given the initial shortage of doses and types of immunizers, the chronic underfunding of the health sector that jeopardized the purchase and distribution of inputs, and the population hesitation to take the vaccines, as identified in several local studies (28–30).

In addition to these factors that weakened the advancement of vaccination coverage, other variants were identified in the country, such as Gamma, which reached the peak of cases in April 2021 and increased the number of deaths in the country, and Omicron, identified in December 2021 – vacation and recess period – which culminated in the loosening of measures and the increase in the number of deaths, albeit to a lesser extent, at that time (31). The appearance of these new variants may have delayed the effectiveness of the vaccines due to the virus mutation process together with the drop in immunity observed a few months after the application of the vaccine schedule (32). This reinforces the importance of maintaining measures to combat and accelerate the immunization of the entire population at that time.

Another aspect worth mentioning is that the introduction of the Omicron variable in the country coincided with an influenza A virus epidemic, which increased the number of cases of severe acute respiratory syndrome (SARS) and hospitalizations, again bringing challenges in testing and data accuracy, as the reception of data by health agencies was interrupted for several weeks, compromising the monitoring and analysis of the evolution of the pandemic (22).

Even though all these problems affected lethality and mortality indicators, as well as the coordinated advancement of vaccination and immunization of the entire population, the study revealed the effectiveness of vaccination in relation to its transmissibility, identified by the low average incidence in places with high vaccination coverage. Moreover, the result of the linear regression showed that places with low vaccination coverage had higher records of new deaths. These findings emphasize the effectiveness of immunization against COVID-19, as identified in several studies (22, 33, 34).

In a study published by COVID-19 Observatory of Oswaldo Cruz Foundation (35), a significant reduction in the incidence of serious hospitalized cases and deaths with COVID-19 confirmation criteria was observed after the start of the National Vaccination Campaign. This indicates that vaccination was an important factor in reducing morbidity and mortality in the country. Furthermore, similar studies that sought to analyze the effectiveness of vaccines and the spread of the virus (36–39) concluded that complete immunization is related to a short duration of viral dissemination and a lower attack rate, lower risk of progression to severe forms of the disease and lower incidences.

The results revealed an increase in vaccination coverage as a reflection of the increase in incidence, suggesting that municipalities with high incidences reinforced their vaccination policy. However, with the descriptive analysis of the data, it was identified that the places with high incidence also had lower averages of actions. Based on this aspect, it is evident that policies to fight COVID-19 implemented together, such as vaccination and actions to combat it, were more effective in controlling the pandemic.

The study demonstrated a significant inequality in the distribution of the number of beds in the ICU and exclusive ward for COVID-19. The distribution of beds was greater in smaller municipalities, with emphasis on Ivaiporã, which also had a higher mean lethality during the study period, Telêmaco Borba, and Guarapuava. Such a finding can be justified by the opening of hospitals dedicated to the exclusive care of the disease in these regions in the second year of the pandemic (40) as a government response plan to regional demands.

Other municipalities that had a high average of ICU beds were Telêmaco Borba, Francisco Beltrão, and Cornélio Procópio, all municipalities with a lower population rate, although these locations did not show low lethality. These results are in line with the summary of social indicators released by the Brazilian Institute of Geography and Statistics (41), which demonstrated that larger municipalities had a greater increase in beds to meet the demand for hospitalization.

Larger municipalities such as Curitiba, Maringá, Londrina, and Apucarana had the highest lethality averages but a low distribution of ICU beds. These findings are worth highlighting, as municipalities such as Curitiba and Foz do Iguaçu had a higher average allocation of resources to face the pandemic, which was also notably heterogeneous.

Additionally, municipalities such as Toledo, Foz do Iguaçu, and Francisco Beltrão, with a high incidence, had the lowest distributions of hospital beds. The results corroborate a study carried out in Ceará, which analyzed the spatial distribution of COVID-19 cases and exclusive therapy beds for the disease and showed the shortage of beds in regions with high levels of contamination (42).

During the course of the pandemic, the southern region was one of those that had the greatest impact on bed occupancy rates (22). Due to this scenario, Paraná had more than 4,700 beds exclusively for treating the disease, approximately 1,900 of which were in the ICU by March 2021 (40).

The results of the present study revealed a global average of 21.5 ward beds (in 100,000 inhabitants) exclusively for COVID-19 among the locations studied in the first two years of the pandemic, with an average of 16.3 ICU beds/100,000, higher than the minimum requirement of 10 ICU beds per 100,000 inhabitants (43). Until January 2021, for example, the rate of ICU beds in Brazil was 9.77/100,000 inhabitants (44), so most municipalities had a higher number of ICU beds dedicated to the disease than the national average.

Research has shown that in the South region, Paraná had the lowest rate of beds available exclusively for COVID-19, although it was one of the states with the greatest distribution compared to the North and Northeast regions of the country, which were highly affected by the disease (44, 45). This reality only bolsters the questioning of the fragility of national coordination in the process of coping with the disease.

Finally, the study provided an analysis of the expenses paid by the municipalities evaluated exclusively for the COVID-19 pandemic. The highest average distribution of resources for disease was identified in the municipality of Curitiba, the capital of the state, and in the city of Foz do Iguaçu. The smallest contributions were allocated to the municipalities of Umuarama and Ponta Grossa.

It is justifiable that the state capital receives the greatest contribution, due to its wealth and economic capital, with the highest Gross Domestic Product (GDP) in Paraná and the 6th largest national GDP. Curitiba was also one of the Brazilian capitals that received the greatest federal support for COVID-19. Foz do Iguaçu, located in a triple border region, considered a major political-economic hub, ranked 6th in Paraná’s ranking of municipalities with the highest GDP and 59th in the national ranking (46, 47). In addition, Curitiba and Foz do Iguaçu had high averages in health indicators, justifying the investment made to face the crisis in these locations.

According to Poiatti and Pedroso (48), places with high incomes have greater ability to apply more stringent and prolonged restrictive measures. Thus, it was expected that those municipalities receiving greater support would also present higher averages of coping actions. However, descriptive data revealed that this did not occur. Furthermore, places with lower average resources but high average actions were observed, such as Cornélio Procópio and Umuarama.

These findings are important since the contribution destined for COVID-19 did not serve exclusively to implement health actions, even though this was the public sector most affected by the disease, but also to implement control policies in the economic, environmental, educational, cultural, security, work, and transportation sectors, among others.

Studies revealed that the contributions destined exclusively for actions to control and mitigate the pandemic in Brazil were small, corresponding to 4.93% of the national GDP. In the United States and the United Kingdom, for example, these expenses represented 25.5 and 19.3% of their local GDP (5, 46). This low amount notably influenced the allocation of resources to the states, as in the case of Paraná, which allocated 0.23% of its local GDP to COVID-19 in 2020 and only 0.15% in 2021 (49), and, consequently, in transfers to municipalities.

With regard specifically to health, the government of Paraná restricted investments for the sector in 2020 and 2021, limiting itself to complying only with the constitutional minimum of 12% of current net revenue (RLC), and the state registered one of the smallest investments in health in the country during the crisis. According to data from the Public Health Budget Information System (SIOPS), Paraná invested a total of BRL 589.28 per inhabitant for health in the first two years of the crisis, with the national average being BRL 634.36 per habitant (50, 51). This fact directly affected municipal political and economic organizations, considering that the adoption of measures to contain the virus was directly related to the implementation of broad economic packages.

This scenario is worrying because by the end of the period evaluated, Paraná was among the five states with the highest coefficients of mortality (370.8/100,000 inhabitants) and incidence (20,879.3/100,000 inhabitants) registered in the country. In addition, during the course of the crisis in the first two years, the state also experienced critical periods of overcrowding in hospitals and ICU beds (22, 52), which made the proper transfer of resources crucial for the implementation of measures to contain COVID-19.

The synthesis of the results presented by the study demonstrated the importance of the organized and targeted implementation of actions and policies to face the pandemic in the state of Paraná, as well as the adequate transfer of exclusive resources to face a health crisis of great repercussion, such as the one caused by COVID-19. It was clear that coping actions applied in conjunction with the expansion of vaccination coverage, in view of the epidemiological scenario throughout the course of the disease, revealed greater effectiveness.

In addition, organized structural response actions, such as the expansion of beds based on the progress of the pandemic and the transfer of resources destined for this purpose, were considered of paramount importance. However, it was observed that the implementation of these policies did not occur homogeneously in the municipalities studied, which may be a reflection of the limited national governance, in view of the initial denialist position of the government at the time that interfered in the local rulers decision-making, especially in the state of Paraná, one of the states most affected by the disease, with high indicators throughout the course of the pandemic.

In Brazil, national governance and coordination were marked by weaknesses in leadership and federative coordination mechanisms. The fragmentation and poor articulation between legal and normative measures at the federal, state and municipal levels compromised the adoption of coping measures, especially harming the economy, employment and social protection. These weaknesses were evident mainly in the availability of insufficient information or without scientific evidence, divergent messages between the national government and its executing units, such as the Ministry of Health, which hampered communication with subnational entities and the population (53).

As a federative country that is organized into three levels of government (Union, State and Municipalities), with an universal, public and national healthcare system, it was expected that the combat of COVID-19 would be based on coherence with its triune structure. In practice, this did not happen. The governance of the Brazilian response was marked by divergent actions and initiatives between federative entities, under weak national coordination and little participation from other sectors (53).

Furthermore, the legislative and normative production of federal entities also suffered from the limitations of the national government. Since the beginning of the pandemic, state and municipal governments have published a series of laws, decrees and ordinances that aimed at controlling the disease, organizing the system and reducing health impacts. However, such fragmentation resulted in overlapping actions, areas discovered by implemented plans, in addition to competition between governments for resources and inputs (53).

In Canada, for example, the strong leadership and coordination capacity between authorities, as well as the adoption of effective communication strategies with the population, enabled the implementation of restriction measures in a timely manner, facilitating the actions of the consolidated public health system local, with a tradition in public health and strong national authority. The reflection of strong governance was also demonstrated by the various measures to support the economy, social protection and workers (54).

Countries such as China and South Korea showed high adherence to control measures, due to strong national governance and coordination between surveillance and testing. The initial response plan was immediate, with isolation and quarantine actions for confirmed cases, enabling rapid containment of the focus of the epidemic. In these countries, the health system was based on investments by national governments in high technologies that enabled the advancement of surveillance, prevention and diagnosis tools (55, 56).

According to Muller and Filho (18), the countries that were most successful in combating the spread of COVID-19 were China, Japan and Southeast Asian countries such as Thailand, Indonesia and Vietnam. This fact can be explained by the fact that these countries promoted immediate initial actions, according to previous experiences, with the adoption of lockdown and rigorous coping measures, in addition to implementing large-scale testing systems and programs.

In Germany and Spain, national governance was marked by strong articulation between surveillance and national and federative coordination. These countries also stood out for their investments in innovative research and practices, regarding the development of genetic tests and mapping, as in the case of Spain, and investments in innovative practices and digital surveillance, as in Germany (57, 58).

On the contrary, failures in alert systems and underreporting of cases were observed at some point in several countries, such as Brazil, Spain, China, Mexico, and impacted the entry and advancement of the disease in these territories. In Mexico, as in Brazil, there was weak national governance, impacting the coordination of measures to protect the economy, employment and social protection. Furthermore, the response plan to face the crisis occurred late. There was ambiguity in measures between different regions, especially at the beginning of the pandemic. As a result, the weight of decisions and coordination of actions fell on state and local administrations which, without adequate national coordination and accountability, ended up competing for resources (53, 59).

## 5 Conclusion

The study highlighted the importance of carrying out a robust analysis of public policies to face emergencies of global importance so that government entities are prepared for future crises of great repercussions, such as the COVID-19 pandemic, and are able to optimize the implementation of actions and measures, as well as the allocation of public resources to meet the immediate needs of the different sectors affected by the disease or injury. For this, political coordination by the central governments is considered fundamental.

The results of the study are intended to serve as a tool for the development of public policies to control infectious diseases that may emerge in a future scenario, both in the health sector and in other political-economic sectors, in the three levels of government, as they demonstrated the effectiveness in combating the advancement of the disease, especially when applied together, in a synchronous and planned manner. For this to be possible, federative entities must align their goals and objectives and implement public policies based on scientific evidence. The same applies to the implementation of prevention policies, such as the vaccination campaign, which proved to be the most effective measure in controlling the once emerging crisis.

Regarding funding for the control of COVID-19, it is believed that the study can serve as an aid for organizing and planning resources in future emergency situations. The need to prioritize financial allocation to areas of greater relevance, such as health and education, was demonstrated by this study. In this sense, the three levels of government (Union, State and Municipalities), when exercising their financial autonomy, must take into account contingencies that may occur.

In the academic field, it is hoped that the results can inspire similar studies in other entities of the federation, including in other countries, to encourage comparative research of different sets of policies and control measures, listing actions by degree of effectiveness. In this sense, as a proposal for future research, the study allows the creation of an instrument for evaluating public policies to combat health crises.

As limitations of the study, the following are highlighted: the novelty of the situation studied resulted in the lack of statistical and methodological indicators for analyzing the study; the scarcity of similar analytical studies in relation to the topic in question; and carrying out research with secondary data.

Regarding this last limitation, care must be taken when working with secondary data, as there are particularities that can compromise the accuracy of the indicators, due to technical, administrative and structural differences in the processing of data between different government entities. However, it should be noted that the data were taken from the Epidemiological Bulletins of SESA/Pr, which are daily updated in the *Portal da Transparência* website. This database represents an official source for measuring and monitoring the indicators of the study population. To mitigate these limitations, data were compared with files made available by the SESA/Pr, which authorized the research and obtained information about the pandemic in the State of Paraná.

## Data availability statement

The original contributions presented in the study are included in the article/Supplementary material, further inquiries can be directed to the corresponding author.

## Author contributions

BB: Conceptualization, Data curation, Formal analysis, Investigation, Methodology, Resources, Software, Visualization, Writing – original draft, Writing – review & editing. BO: Conceptualization, Formal analysis, Funding acquisition, Methodology, Project administration, Resources, Supervision, Visualization, Writing – review & editing. LR: Conceptualization, Data curation, Investigation, Methodology, Resources, Visualization, Writing – review & editing. JJ: Data curation, Formal analysis, Methodology, Resources, Software, Visualization, Writing – review & editing.
