# Supplementary material for: Analysis of public policies to combat COVID-19 in the state of Paraná, Brazil
Source: Front Public Health. 2024 Jul 17;12:1384561. doi: 10.3389/fpubh.2024.1384561 (PMC11288802; doi:10.3389/fpubh.2024.1384561)
Supplement: Supplementary file 1 [file Table_1.DOCX]

Table 1- Global average of the variables analyzed over 24 months (March 2020 to March 2022) in the 22 municipalities hosting the Health Regions of the State of Paraná, Brazil.

| **Cities** | **HOSPB** | **ICUB** | **Actions** | **EXP1** | **VC** | **NC** | **ND** | **LC** | **MC** | **IF** | **PHENC** | **TC** | **SMG** | **SLC** | **MASK** | **TEL** | **CUR** |
| --- | --- | --- | --- | --- | --- | --- | --- | --- | --- | --- | --- | --- | --- | --- | --- | --- | --- |
| Apucarana | 32.4 | 12.9 | 4.6 | 1668908.7 | 39.4 | 558.0 | 16.5 | 4.3 | 175.2 | 4760.0 | 1.0 | 0.2 | 0.5 | 0.6 | 1.0 | 1.0 | 0.4 |
| Campo Mourao | 15.4 | 19.4 | 5.0 | 2006443.1 | 40.1 | 556.5 | 10.6 | 2.6 | 177.9 | 9456.4 | 1.0 | 0.2 | 0.6 | 0.8 | 1.0 | 1.0 | 0.6 |
| Cascavel | 13.0 | 22.0 | 4.5 | 297205.7 | 38.6 | 3010.0 | 48.3 | 2.3 | 170.2 | 8740.7 | 1.0 | 0.2 | 0.4 | 0.4 | 1.0 | 1.0 | 0.6 |
| Cianorte | 11.5 | 6.1 | 5.1 | 1156345.8 | 37.5 | 780.3 | 7.8 | 1.6 | 112.0 | 7455.6 | 1.0 | 0.2 | 0.7 | 0.7 | 1.0 | 1.0 | 0.5 |
| Cornelio Procópio | 26.8 | 22.5 | 5.0 | 81526.7 | 41.7 | 510.1 | 7.3 | 2.4 | 183.6 | 9241.8 | 1.0 | 0.2 | 0.6 | 0.7 | 1.0 | 1.0 | 0.6 |
| Curitiba | 21.5 | 16.4 | 4.9 | 3239946.3 | 43.0 | 9839.8 | 326.5 | 3.3 | 206.5 | 6237.6 | 1.0 | 0.2 | 0.7 | 0.7 | 1.0 | 1.0 | 0.4 |
| Foz do Iguacu | 22.0 | 21.6 | 4.4 | 2184728.2 | 41.9 | 2970.9 | 50.3 | 2.0 | 240.1 | 11345.4 | 1.0 | 0.2 | 0.5 | 0.6 | 1.0 | 0.8 | 0.2 |
| Francisco Beltrão | 7.5 | 22.6 | 4.4 | 1589435.2 | 40.5 | 1170.0 | 11.8 | 1.7 | 153.4 | 11050.7 | 1.0 | 0.4 | 0.5 | 0.5 | 1.0 | 1.0 | 0.2 |
| Guarapuava | 42.0 | 19.2 | 4.9 | 404761.7 | 35.0 | 1550.2 | 26.0 | 1.6 | 158.2 | 7391.3 | 1.0 | 0.2 | 0.8 | 0.5 | 1.0 | 1.0 | 0.5 |
| Irati | 13.3 | 13.1 | 5.2 | 816864.2 | 38.1 | 650.3 | 8.3 | 1.6 | 146.2 | 8702.3 | 1.0 | 0.1 | 0.7 | 0.7 | 1.0 | 1.0 | 0.7 |
| Ivaiporã | 134.3 | 63.3 | 4.4 | 561701.1 | 46.0 | 262.5 | 4.0 | 4.0 | 157.4 | 7264.3 | 1.0 | 0.2 | 0.5 | 0.5 | 1.0 | 1.0 | 0.2 |
| Jacarezinho | 24.8 | 7.1 | 5.1 | 815007.5 | 38.3 | 424.6 | 5.1 | 1.1 | 138.9 | 9501.9 | 1.0 | 0.2 | 0.8 | 0.7 | 1.0 | 1.0 | 0.4 |
| Londrina | 21.5 | 18.8 | 4.7 | 1637353.9 | 38.2 | 5765.6 | 95.9 | 2.8 | 190.3 | 9285.2 | 1.0 | 0.2 | 0.6 | 0.7 | 1.0 | 1.0 | 0.3 |
| Maringa | 22.9 | 16.3 | 5.2 | 1268207.3 | 46.9 | 4624.5 | 69.4 | 3.1 | 191.2 | 9338.6 | 1.0 | 0.2 | 0.6 | 0.7 | 1.0 | 1.0 | 0.8 |
| Paranaguá | 14.4 | 15.8 | 5.2 | 583866.7 | 38.6 | 1188.2 | 23.7 | 2.6 | 190.6 | 9009.8 | 1.0 | 0.3 | 0.6 | 0.7 | 1.0 | 1.0 | 0.7 |
| Paranavaí | 19.2 | 15.2 | 4.4 | 555048.3 | 39.7 | 895.7 | 13.3 | 2.5 | 164.7 | 7917.1 | 1.0 | 0.2 | 0.4 | 0.7 | 1.0 | 0.8 | 0.4 |
| Pato Branco | 23.5 | 9.7 | 5.1 | 1756728.3 | 41.2 | 999.2 | 12.8 | 2.0 | 174.8 | 9436.7 | 1.0 | 0.2 | 0.6 | 0.8 | 1.0 | 1.0 | 0.6 |
| Ponta Grossa | 12.5 | 10.0 | 4.7 | 24875.2 | 33.3 | 3205.5 | 58.8 | 1.9 | 189.1 | 8338.7 | 1.0 | 0.2 | 0.4 | 0.6 | 0.9 | 1.0 | 0.7 |
| Telêmaco Borba | 38.6 | 39.4 | 4.8 | 530379.6 | 38.6 | 999.3 | 16.2 | 1.8 | 246.0 | 12794.3 | 1.0 | 0.3 | 0.7 | 0.7 | 1.0 | 1.0 | 0.2 |
| Toledo | 0.0 | 15.2 | 4.6 | 864262.3 | 35.4 | 1695.5 | 19.5 | 1.3 | 166.7 | 11487.9 | 1.0 | 0.2 | 0.5 | 0.5 | 0.9 | 1.0 | 0.5 |
| Umuarama | 34.9 | 19.6 | 5.1 | 27898.9 | 36.7 | 1406.9 | 14.8 | 2.1 | 145.6 | 9977.9 | 1.0 | 0.2 | 0.6 | 0.7 | 1.0 | 1.0 | 0.7 |
| União da Vitória | 20.5 | 12.2 | 5.0 | 572208.8 | 34.8 | 520.2 | 6.2 | 1.3 | 115.8 | 7534.1 | 1.0 | 0.1 | 0.6 | 0.7 | 1.0 | 1.0 | 0.7 |
| **Average** | **21.5** | **16.3** | **4.9** | **815935.9** | **38.6** | **1084.6** | **15.5** | **2.1** | **172.5** | **9125.8** | **1.0** | **0.2** | **0.6** | **0.7** | **1.0** | **1.0** | **0.5** |
| **Standard Deviation** | **25.0** | **11.5** | **0.3** | **779334.5** | **3.2** | **2180.0** | **65.8** | **0.8** | **31.5** | **1739.2** | **0.0** | **0.1** | **0.1** | **0.1** | **0.0** | **0.0** | **0.2** |

Source: Research database, 2023.
