# Supplementary material for: Analysis of public policies to combat COVID-19 in the state of Paraná, Brazil
Source: Front Public Health. 2024 Jul 17;12:1384561. doi: 10.3389/fpubh.2024.1384561 (PMC11288802; doi:10.3389/fpubh.2024.1384561)
Supplement: Supplementary file 2 [file Table_2.DOCX]

Table 2- Correlation Matrix between the variables analyzed in the 22 municipalities hosting HRs in the State of Paraná over 24 months.

|  | **HOSPB** | **ICUB** | **Actions** | **EXP** | **VC** | **LC** | **MC** | **IR** | **NC** | **ND** | **EXP1** | **BC** | **SMG** | **SLC** | **MASK** | **TEL** | **CUR** |
| --- | --- | --- | --- | --- | --- | --- | --- | --- | --- | --- | --- | --- | --- | --- | --- | --- | --- |
| **HOSPB** | 1* | 0.36 | 0.18 | -0.12 | 0.07 | 0.22 | 0.24 | -0.14 | -0.04 | 0 | 0.01 | -0.19 | 0.39 | 0.23 | 0.47* | -0.05 | -0.12 |
| **ICUB** |  | 1* | -0.3 | -0.07 | 0.18 | 0.07 | 0.64* | 0.56* | 0.06 | 0.02 | 0 | 0.5* | -0.14 | -0.13 | 0.2 | -0.08 | -0.45* |
| **Actions** |  |  | 1* | -0.13 | 0.05 | -0.11 | -0.31 | -0.12 | -0.13 | -0.07 | -0.02 | -0.39 | 0.67* | 0.6* | 0.21 | 0.54* | 0.67* |
| **EXP** |  |  |  | 1* | 0.55* | 0.42 | 0.3 | -0.15 | 0.55* | 0.61* | 0.68* | 0.28 | 0.12 | 0.11 | 0.24 | -0.19 | -0.41 |
| **VC** |  |  |  |  | 1* | 0.52* | 0.41 | 0.06 | 0.35 | 0.34 | 0.37 | 0.28 | -0.03 | 0.25 | 0.49* | -0.22 | -0.14 |
| **LC** |  |  |  |  |  | 1* | 0.37 | -0.5* | 0.4 | 0.41 | 0.38 | 0.1 | -0.29 | 0.07 | 0.25 | -0.09 | -0.05 |
| **MC** |  |  |  |  |  |  | 1* | 0.37 | 0.41 | 0.36 | 0.29 | 0.46* | -0.23 | 0.04 | -0.05 | -0.29 | -0.39 |
| **IR** |  |  |  |  |  |  |  | 1* | -0.18 | -0.3 | -0.32 | 0.42 | -0.05 | -0.01 | -0.16 | -0.07 | -0.24 |
| **NC** |  |  |  |  |  |  |  |  | 1* | 0.94* | 0.85* | 0.17 | -0.02 | -0.06 | -0.06 | -0.12 | -0.16 |
| **ND** |  |  |  |  |  |  |  |  |  | 1* | 0.97* | 0.18 | 0.07 | 0.01 | 0 | -0.12 | -0.18 |
| **EXP1** |  |  |  |  |  |  |  |  |  |  | 1* | 0.19 | 0.17 | 0.07 | 0.09 | -0.13 | -0.22 |
| **BC** |  |  |  |  |  |  |  |  |  |  |  | 1* | -0.21 | -0.39 | -0.04 | -0.05 | -0.54* |
| **SMG** |  |  |  |  |  |  |  |  |  |  |  |  | 1* | 0.39 | 0.32 | 0.38 | 0.03 |
| **SLC** |  |  |  |  |  |  |  |  |  |  |  |  |  | 1* | 0.37 | -0.05 | 0.18 |
| **MASK** |  |  |  |  |  |  |  |  |  |  |  |  |  |  | 1* | -0.12 | -0.18 |
| **TEL** |  |  |  |  |  |  |  |  |  |  |  |  |  |  |  | 1* | 0.34 |
| **CUR** |  |  |  |  |  |  |  |  |  |  |  |  |  |  |  |  | 1* |

* Statistically significant correlation with a significance level of 5%.

Source: Prepared by the authors, 2023.
